# Successful treatment of adult cecorectal intussusception caused by cecum cancer with mobile cecum: a case report

**DOI:** 10.1186/s40792-021-01180-0

**Published:** 2021-04-15

**Authors:** Hayato Shimoyama, Kimihiko Ueno, Masahiro Samizo

**Affiliations:** 1 Department of Surgery, Kobe Rosai Hospital, 4-2-23 Kagoikedori, Chuouku, Kobe, Hyogo 651-0053 Japan; 2 Department of Gastroenterological Surgery, Toranomon Hospital, 2-2-2 Toranomon, Minatoku, Tokyo 105-8470 Japan; 3 Department of Gastroenterological Surgery, National Hospital Organization Kobe Medical Center, 3-1-1, Nishiochiai, Sumaku, Kobe, Hyogo 654-0155 Japan; 4 Department of Surgery, Sanda City Hospital, 3-1-1 Keyakidai, Sanda, Hyogo 669-1321 Japan

**Keywords:** Cecorectal intussusception, Mobile cecum, Combined transabdominal and trans-anal procedure

## Abstract

**Background:**

Intussusception occurs when a segment of the bowel (the intussusceptum) telescopes into an adjacent segment (the intussuscipiens). Adult intussusception occurs rarely and often requires surgical resection for its treatment. We describe the case of an adult patient with extremely rare cecorectal intussusception treated using a novel combined transabdominal and trans-anal approach, which has not yet been reported in the literature.

**Case presentation:**

A 71-year-old woman was transferred to our hospital for the treatment of upper abdominal pain. Physical examination, laboratory tests, and imaging inspections showed strangulated bowel obstruction induced by intussusception associated with the intra-rectal mass. We performed an emergency operation and treated the intussusception using a combined transabdominal and trans-anal approach. The intraoperative findings revealed bloody ascites and a potentially malignant tumor that had moved toward the anal side from peritoneal reflection. The tumor served as the lead point in the cecum with mobile cecum. After reducing the intussusception using the combined procedure, we removed the ileocecal portion. The intraoperative and histopathological findings suggested that cecum cancer with mobile cecum had caused the cecorectal intussusception. The patient had an uneventful postoperative course, except for postoperative pulmonary pneumonia.

**Conclusion:**

To the best of our knowledge, this is the first reported case of adult cecorectal intussusception due to cecum cancer with mobile cecum successfully treated using the combined transabdominal and trans-anal approach. This combined procedure may be useful in treating the intussusception where the lead point is distal from the peritoneal reflection.

## Background

Adult intussusception is rare, accounting for only 5% of all cases that often need surgical resection due to the risk of intestinal necrosis or malignancy [1].

Of all the adult intussusceptions, 38–44% have occurred in the colon and 52–55% in the small intestine [2]. Adult colonic intussusception mostly occurs in the sigmoid colon, transverse colon, and the cecum [3]. Cecorectal intussusception is extremely rare.

To the best of our knowledge, this is the first report of adult cecorectal intussusception caused by cecum cancer with mobile cecum. The intussusception was managed using a novel combined transabdominal and trans-anal procedure for the first time. We herein present a case of cecorectal intussusception due to cecum cancer with mobile cecum.

## Case presentation

A 71-year-old woman with chronic kidney failure was transferred to our hospital to treat upper abdominal pain that persisted for 4 days. The patient was conscious, but visibly distressed. Her body temperature, blood pressure, heart rate, and respiratory rate were 36.3 °C, 167/68 mmHg, 82 bpm, and 28 bpm, respectively. Physical examination revealed diminished bowel sounds, lower abdominal distension, and rebound tenderness around the umbilicus. Her laboratory investigations were unremarkable (creatinine phosphokinase 53 U/l, lactate dehydrogenase 212 IU/l) except for significant kidney failure with a creatinine level of 7.73 mg/dl, blood urea nitrogen level of 48.6 mg/dl, and mild elevation of C-reactive protein (0.5 mg/dl). Arterial blood gas analysis revealed a low pH of 7.30, partial pressure of carbon dioxide 32.9 mmHg, and severe metabolic acidosis with a bicarbonate ion level of 16.0 mmol/l, a base excess of − 9.1 mmol/l, and lactate level at 0.7 mmol/l under room air atmosphere.

**Fig. 1:**
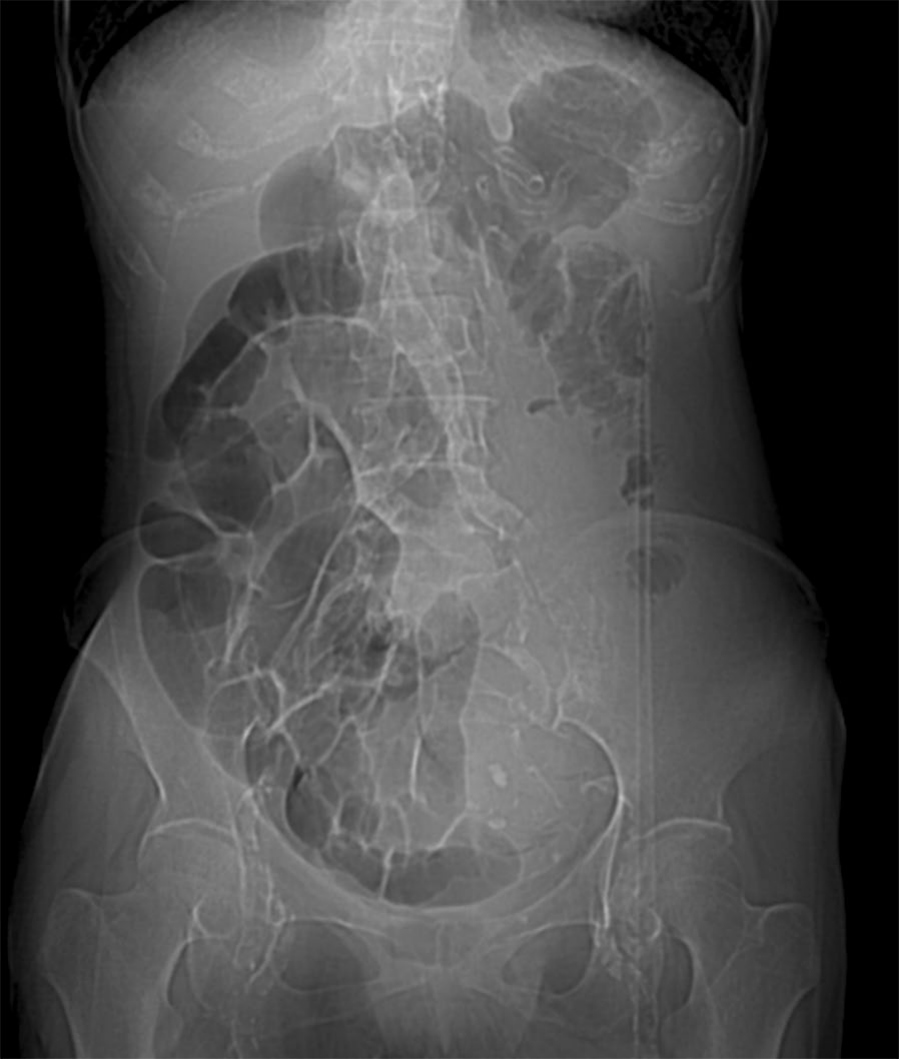
Abdominal computed tomography (CT) scout. Findings suggest intestinal dilation and intestinal gas-like obstruction on the right side and a paucity of gas in the middle to the left side

**Fig. 2:**
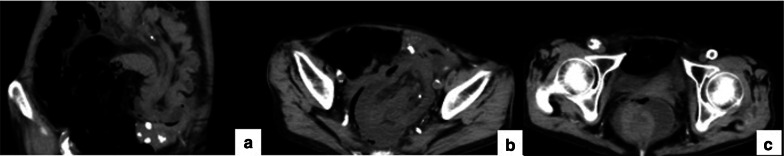
Abdominal computed tomography (CT) images. **a** Sausage-shaped sign in the descending colon. **b** Enlargement of the colorectal wall, fat within the intussusception, and air within the intussuscipiens in the pelvis. **c** Mass in the rectum

A computed tomography (CT) scout image showed distension in the right abdomen (Fig. 1). Abdominal CT scan revealed ascites around the liver and the spleen, small bowel extension, sausage-shaped sign from the transverse colon to the rectum, and a mass (40 mm in diameter) in the rectum (Fig. 2). Based on these findings, we diagnosed the patient with a strangulated bowel obstruction induced by intussusception associated with the intra-rectal mass and subsequently performed emergency surgery.

**Fig. 3:**
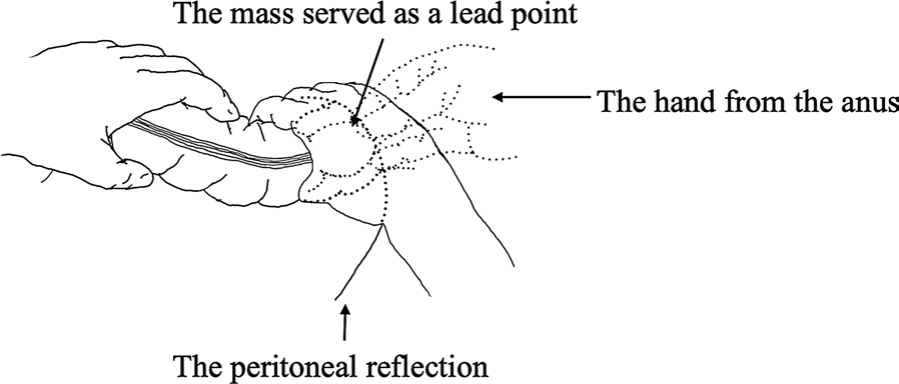
Combined transabdominal and trans-anal procedure. We pushed the mass, which is located distal of the peritoneal reflection, through the anus to the oral side. Subsequently, we used the Hutchinson maneuver

**Fig. 4:**
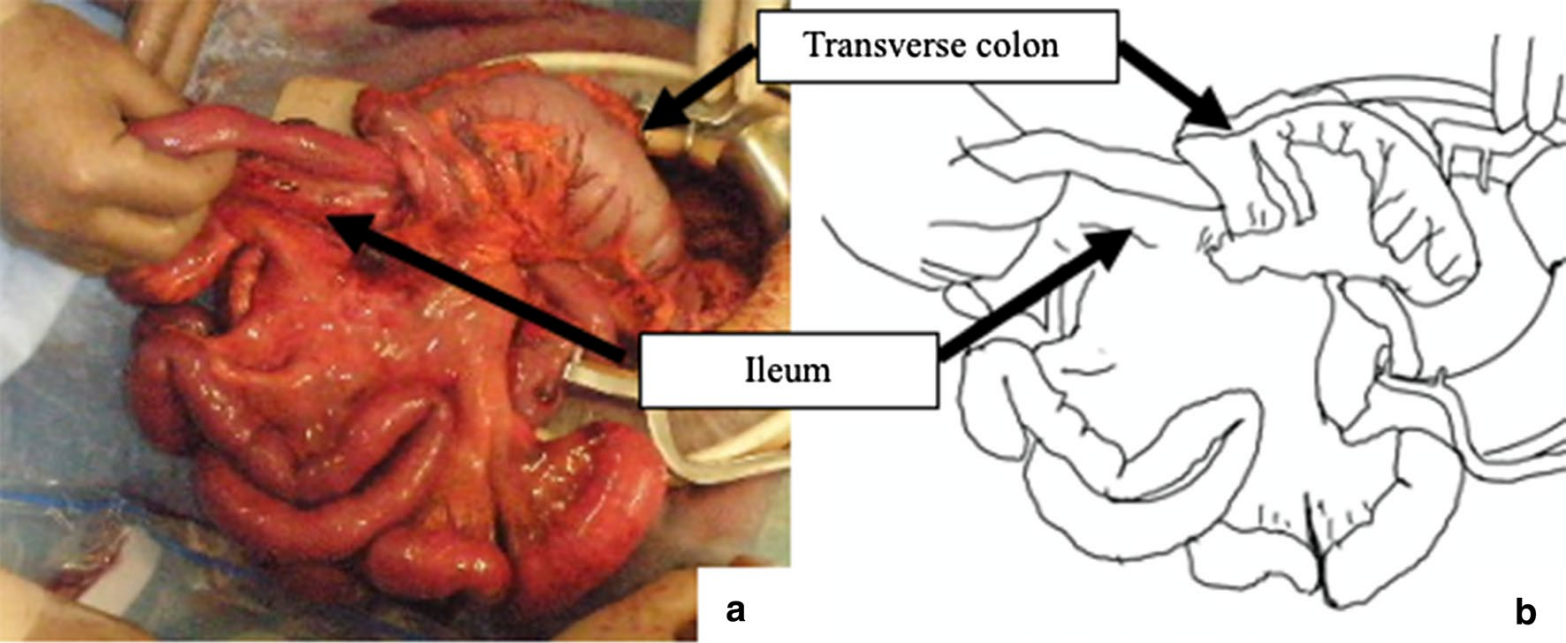
Intraoperative view. Ascending colon and cecum are invaginated in the transverse colon with the mobile cecum. **a** Intraoperative photograph. **b** Schematic representation

Laparotomy was performed with the patient in the lithotomy position under general anesthesia. We found bloody ascites and intussusception. The mass served as a lead point and was moved to the anal side by peritoneal reflection. We were unable to relieve this invagination by the Hutchinson maneuver alone. Therefore, we performed the trans-anal procedure to push the mass gently through the anus to the oral side. Subsequently, we released the invagination using the Hutchinson maneuver (Fig. 3). Although the bowel was not necrotic, we found a potentially malignant tumor in the cecum with mobile cecum, which was considered to be the lead point of this intussusception (Fig. 4). We found that the regional lymph nodes were not swollen and that the tumor moved well against the intestinal wall, indicating that it had not invaded the muscularis propria. There was no right colic artery and vein arising from the superior mesenteric artery or vein. We cut the roots of the ileocecal artery and vein and resected the ascending colon and the ileocecal portion with D2 lymph node dissection as the formal oncologic treatment. The intestine was then reconstructed by functional end-to-end anastomosis. All the procedures were successfully performed without any adverse events.

**Fig. 5:**
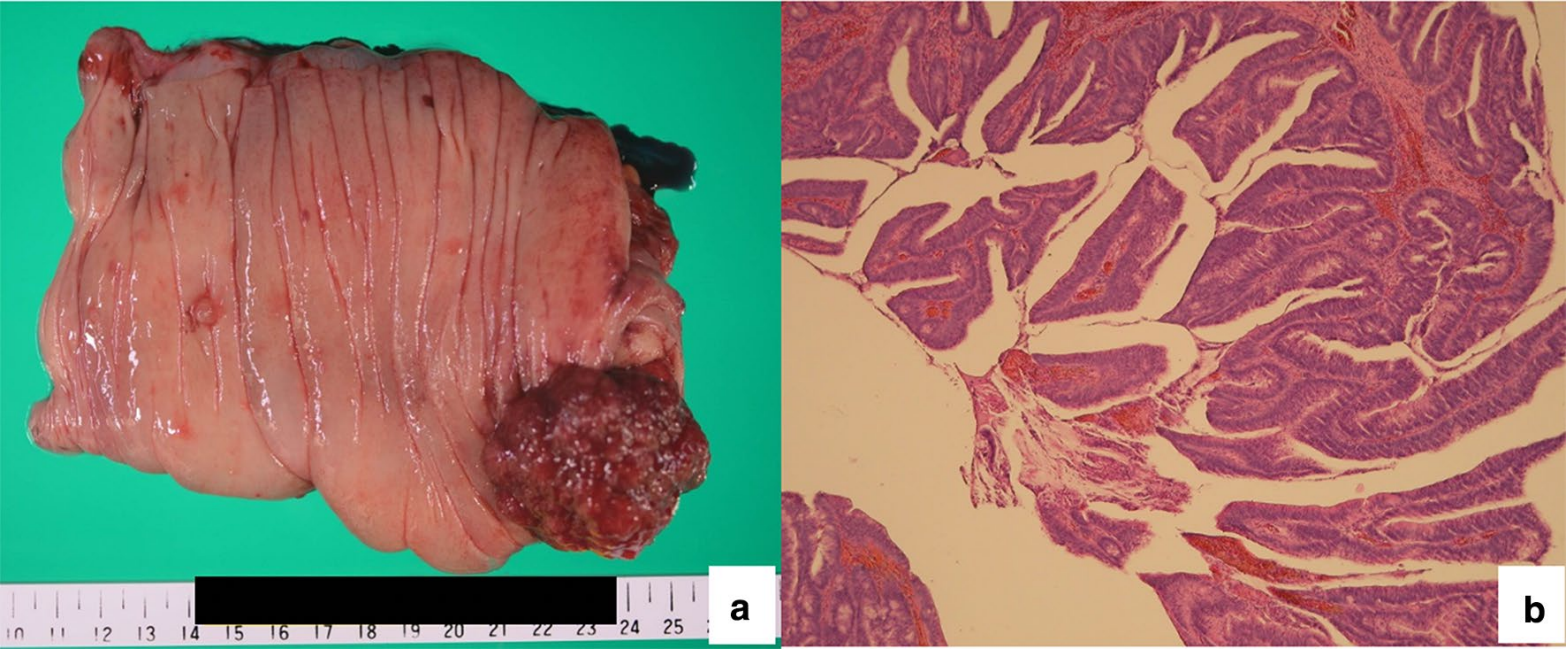
Resected specimen and histopathological images. **a** The tumor is reddish, round, elastic soft, granular surfaced, measuring 45 mm in length and 40 mm in width in the cecum. **b** The lesion is stained with hematoxylin and eosin (magnification: 200×)

The resected mass was reddish, round, elastic, and soft, with a granular surface measuring 45 mm in length and 40 mm in width in the cecum (Fig. 5a). Histopathological examination of the specimen revealed a moderately to well-differentiated tubular adenocarcinoma in the tubulovillous adenoma (Fig. 5b). The cancerous lesion was found superficially in the mucosal layer with no regional lymph node metastasis.

The cecorectal intussusception seemed to have occurred secondary to the cecum cancer with mobile cecum based on the intraoperative and histopathological findings. The patient had an uneventful postoperative course, except postoperative pneumonia, and was discharged from the hospital on the 25th postoperative day after an antibiotic infusion therapy. No recurrence of cecum cancer was identified 3 years after the surgery.

## Discussion

Intussusception occurs when a segment of the bowel (the intussusceptum) telescopes into an adjacent segment (the intussuscipiens). Intussusception is often encountered in infants and children, and only 5% of all cases have been reported to occur in adults [1]. Children with intussusception present with acute-onset colicky pain, vomiting, and bloody mucoid stools, described as “currant jelly stools”. In contrast, adults rarely exhibit these symptoms. The clinical presentation in adult intussusception is often chronic, and most patients present with nonspecific symptoms suggestive of intestinal obstruction. Therefore, the clinical diagnosis can be challenging in adults. The etiology of adult intussusception can be due to idiopathic, benign, or malignant processes; however, approximately 65% of adult intussusception is related to malignant lesions [4]. Although adult intussusception mostly occurs in the sigmoid colon, transverse colon, or cecum [3], cecorectal intussusception is extremely rare. To the best of our knowledge, there are only four reports of cecorectal or cecoanal intussusceptions in the literature (Table 1). We present the first report of adult cecorectal intussusception due to cecum cancer with mobile cecum.

**Table 1:** Reports of cecorectal or cecoanal intussusception

Case	Author	Sex/age	Chief complaint	Diagnostic modality	Type of intussusception	Relief invagination	Surgical intervention	Pathological diagnosis
1	Greif et al	M/4	Abdominal pain, prolapse	Not specified	Cecoanal	Unsuccessful	Cecal resection	Burkitt’s lymphoma
2	Shinoda et al	F/29	Diarrhea, vomiting, abdominal pain	Barium enema, colonoscopy	Cecoanal	Manual, enema	Cecal resection	Well-differentiated adenocarcinoma in villous adenoma
3	Suddiqui et al	M/90	Vomiting, abdominal pain	CT	Cecoanal	Not specified	Subtotal colectomy	Villotubular adenoma
4	Chen et al	M/13	Abdominal pain, prolapse	CT	Cecorectal	Not specified	Ileocecal resection	Hematoma
5	Our case	F/71	Upper abdominal pain	CT	Cecorectal	Manual	Ileocecal resection	Well-differentiated tubular adenocarcinoma in the tubulovillous adenoma

In our patient, the mobile cecum enabled cecum cancer to reach the rectum. The mobile cecum is characterized by abnormal mobility of the cecum and ascending colon due to the failure of the cecum and right colon to fuse with the posterior parietal peritoneal wall [5]. Although abnormal mobility of the cecum and ascending colon are present in 10–20% of the population, colonic intussusception related to the mobile cecum is rare in adults [6–9]. Moreover, cecum cancer with mobile cecum may cause cecorectal intussusception.

The clinical examination of such cases may include radiography, ultrasound, CT scan of the abdomen, barium enema, and colonoscopy [10, 11]. The plain abdominal radiography is rarely diagnostic and often demonstrates nonspecific signs of intestinal obstruction [12]. Barium enema shows the intussusception as an intraluminal crescent or round-filling defect [13]. A barium enema is often used as a therapeutic procedure for intussusception in children. The ultrasound appearance of a typical intussusception is highly characteristic and has been well described in the literature. A peripheral hypoechoic ring with central echogenicity, known as the target sign (in transverse view) and pseudokidney sign (in longitudinal view), corresponds to the bowel wall surrounding the hyperechoic mesenteric fat contained within the intussusception [14]. While ultrasound carries no radiation risks and is readily available, in our opinion, this mode of examination is operator-dependent and requires an experienced examiner.

Colonoscopy is most useful in adult intussusception involving the colon and the terminal ileum and cecum as it shows the location and allows biopsy to aid the diagnosis and surgical plan [15, 16]. Colonoscopy should be avoided in patients with the possibility of perforation or ischemia [17]. A CT scan may show intussusception as a sausage-shaped mass in the longitudinal axis, and as a target mass in the transverse axis. Abdominal CT scan has been reported to be the most useful imaging modality [18].

Although barium enema and colonoscopy can reduce intussusception, many studies recommended laparotomy due to the high incidence of underlying malignancy in colonic intussusception and the inability to differentiate non-operatively the benign and malignant causes of enteric intussusceptions [19].

Pre-operative barium enema, which is the first-line therapy in pediatric intussusception, should not be performed in adults [1, 20, 21]. As a technical matter, the decision to reduce the intussusceptions before resection or resect the bowel en bloc should be made by the surgeon at the time of the laparotomy based on the location, character of the bowel, presence or absence of ischemia, proper oncologic principles, and risk assessment of possible seeding of the tumor cells at the time of reduction [19].

## Conclusion

We presented a case of adult cecorectal intussusception due to cecum cancer with mobile cecum that was successfully treated using a combined transabdominal and trans-anal procedure. This combined procedure may be useful in treating the intussusception where the lead point is distal from the peritoneal reflection.

## Data Availability

Not applicable.

## Abbreviations

CT: Computed tomography
US: Ultrasound sonography
